# Low-Energy Single-Electron Detector with Submicron Resolution

**DOI:** 10.1021/acsphotonics.5c02404

**Published:** 2025-12-21

**Authors:** Luis Alfredo Ixquiac Méndez, Martino Zanetti, Tilman Kraeft, Thomas Juffmann

**Affiliations:** † 27258 University of Vienna, Faculty of Physics, VCQ, 1090 Vienna, Austria; ‡ 360818 University of Vienna, Max Perutz Labs, 1030 Vienna, Austria

**Keywords:** Single-electron detection, low-energy electron detector, scintillator-based detector, YAG, Ce scintillator, high-resolution electron detection, electron diffraction at atmospheric pressure, Air-SEM

## Abstract

Single-electron detectors are a key component of electron microscopes and advanced electron optics experiments. We present a scintillator-based single-electron detector with an estimated spatial resolution of 0.9 μm at an electron energy of 17 keV. Single-electron detection events are identified with an efficiency and purity larger than 0.8 at an electron energy of 17 keV, reaching 0.96 at 30 keV. We show that the detector enables electron diffraction studies with a sample–detector distance comparable to the mean free path of electrons at atmospheric pressure, potentially enabling atmospheric electron diffraction studies.

## Graphical abstract

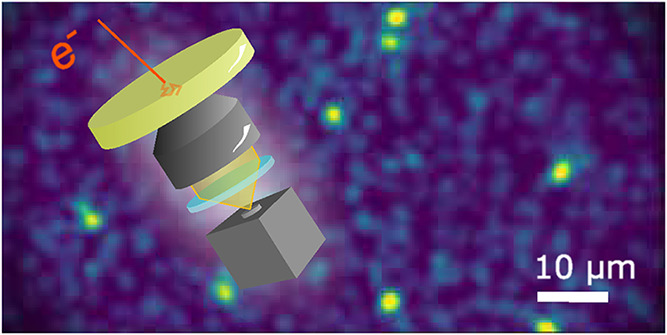

## Introduction

Electron detectors are a crucial component of electron microscopes, electron spectroscopy setups, and quantum electron optics experiments. High spatial and temporal resolution, low noise, and single electron detection efficiency are among the key features of modern electron detectors.

The high sensitivity and speed of direct electron detectors (DED)^1^ have revolutionized dose-sensitive applications such as cryo-electron microscopy or tomography,^2^ and DEDs are now the gold standard in most electron microscopy and spectroscopy applications.^3^ At low electron energies typical for scanning electron microscopy (SEM, 1 to 30 keV), DEDs are often employed in the form of hybrid array detectors. While they enable single electron detection, their spatial resolution is limited by their pixel size which is typically 55 μm^4^ or larger.^5^ Monolithic active pixel sensors offer a pixel size down to 5 μm^6–8^ and have been employed at energies down to 4 keV.^7^ While they are commercially available, they are often prohibitively expensive.

Here, we demonstrate single electron detection and counting based on an Yttrium Aluminum Garnet scintillator doped with Cerium (YAG:Ce^9^) that is imaged with an optical microscope. Using a high numerical aperture objective, we collect an average of 26 photons per 30 keV electron, yielding an efficiency and purity in classifying single-electron events of 0.96. We demonstrate single electron detection in an energy range between 17 and 30 keV, obtaining a spatial resolution estimate of 0.9 μm (2.3 μm) at 17 keV (30 keV), respectively. This is 5× better than state-of-the-art direct electron detectors at the same electron energy. Finally, we show that our new detector enables electron diffraction studies at submm distances between the sample and the screen. This potentially enables miniature diffraction and spectroscopy setups, as well as diffraction studies at atmospheric pressure, avoiding the need for transferring the samples into vacuum.

## Experimental Section

### Setup

The new detector is sketched in Figure 1 (a). Electrons from a modified FEI XL30 Scanning Electron Microscope (SEM) hit a 200 μm thin YAG:Ce scintillator. The electrons deposit energy in the material leading to scintillation light at wavelengths around λ = 550 nm. To enable high-efficiency light-collection, the scintillator is imaged from behind using an oil-immersion objective of high numerical aperture (Olympus 40X UPlanXApo, NA = 1.4). Similar to the design in,^10^ the scintillator serves as a window for the vacuum chamber, allowing the objective to be mounted in air. The oil immersion (*n*_oil_ = 1.51) is crucial as it significantly increases the critical angle beyond which light is trapped within the scintillator material (*n*_s_ = 1.82). To further increase light collection efficiency, the scintillator is coated with a 25 nm Aluminum layer on the vacuum side, which acts as a mirror for the scintillation light (reflectance *R* = 0.84 at λ = 550 nm^11^). Overall, we expect a light collection efficiency of η_L_ = 0.38, assuming isotropic emission from the scintillator (see the Supporting Information (SI)).

**Figure 1:**
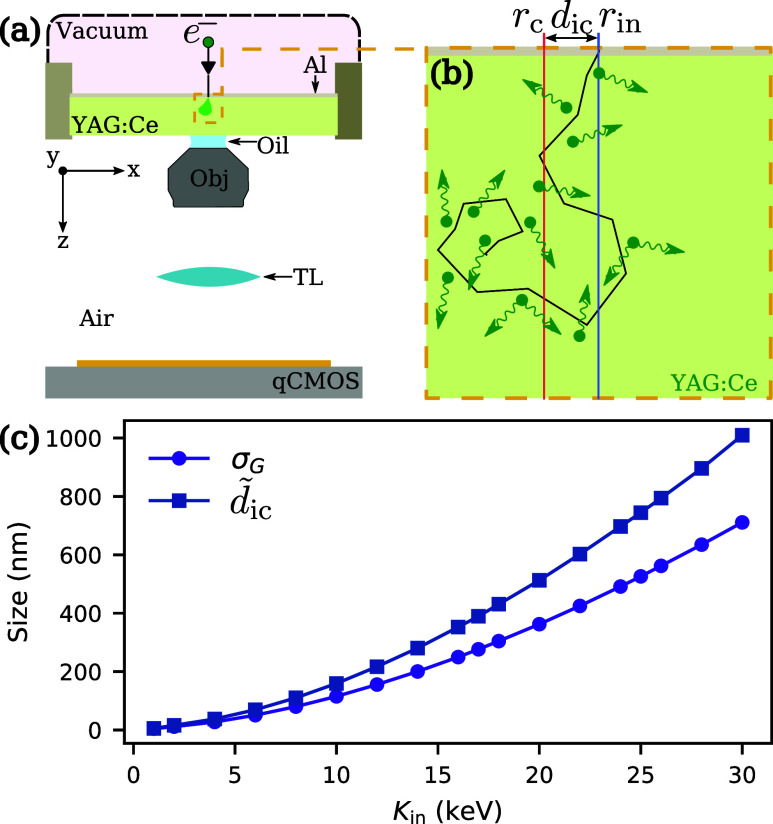
(a) Schematic of the setup: an incident electron e^–^ hits the YAG:Ce scintillator, where a part of its kinetic energy is converted into luminescent photon emission. The resulting signal is relayed by an infinity-corrected optical system onto the CMOS camera sensor. (b) Zoomed-in illustration of the electron trajectory in the YAG:Ce scintillator (black solid line) and the emitted photons (green arrows): *r*_in_ and *r*_c_ denote the coordinates of the electron incidence point and of the center of deposited energy, respectively. The distance between them is *d*_ic_. (c) Radius σ_*G*_ enclosing 68% of the emitted photons (purple line, round markers), and median distance *d̃*_ic_ between *r*_in_ and *r*_c_ (blue line, square markers), both shown as a function of the electrons’ initial kinetic energy.

The objective and a tube lens (Thorlabs AC254–050-A-ML) form an infinity-corrected system that images the scintillation light onto a CMOS camera (Hamamatsu Orca Quest, pixel width 4.6 μm) at a measured effective magnification of *M* = 11.4×. To minimize read noise, the camera is cooled to −34 °C, and operated in photon number resolving mode, yielding a read noise of 0.13 counts rms (see the SI).

### Simulation

When an electron hits the scintillator, it deposits its kinetic energy through multiple collisions,^12,13^ leading to a random electron trajectory as sketched in Figure 1b, and to the emission of scintillation light facilitated by the Ce dopants.^14,15^

We perform Monte Carlo simulations using the CASINO simulation software^16^ to better understand the consequences of these random trajectories on our detector (see the SI). We first calculate the transverse radius σ_*G*_ into which 68% of the energy is deposited, which sets a lower bound on the width of the point-spread function of our detector. The purple line in Figure 1c shows σ_*G*_ as a function of the kinetic energy *K*_in_ of the incoming electron. We see that it increases with *K*_in_, but it remains below 1 μm for energies below 30 keV. Next, we simulate the median distance *d̃*_ic_ between the transverse position *r*_in_ at which the electron enters the scintillator and the center of deposited energy *r*_c_ (blue line in Figure 1c). Again, we see a nonlinear increase with *K*_in_ with a maximum value of *d̃*_ic_ = 1009 nm at 30 keV. This distance limits the accuracy for localizing single-electron detection events.

## Results

### Detection of Single 30 keV Electrons

Figure 2a shows raw data for the detection of 30 keV electrons. While read noise leads to a random distribution of single photon counts, the electron beam induces localized detection events. To identify these events, we first subtract an averaged background image from the raw data, which we recorded with the electron beam off. We then apply a Gaussian filter of radius σ_G_, yielding the image shown in Figure 2b, which shows distinct event detection candidates (details in the SI). To decide which of them correspond to single-electron detection events, we identify the local maxima in the image and calculate the total number of detected photons Σ_ph_ within a circle of diameter *d*_ref_ = 5.2 μm (corresponding to 13 pixels) centered at each maximum. This choice of *d*_ref_ maximizes the *F*_1_-score (defined later), representing an optimal compromise between maximizing signal, and minimizing read-noise. We ignore spurious events and events close to dead pixels of the camera (details in the SI). This yields the histogram in Figure 2d, which shows two distinct peaks. The one to the left, at lower values of Σ_ph_, is due to read noise and is also present in individual background frames (see the SI). Empirically, we find that it can be fitted with a log-normal distribution. The one at higher values of Σ_ph_ corresponds to single-electron detection events. It can be fitted with a normal distribution with a mean photon number of $\overset{®}{\Sigma_{\mathrm{ph}}}=26$ .

**Figure 2:**
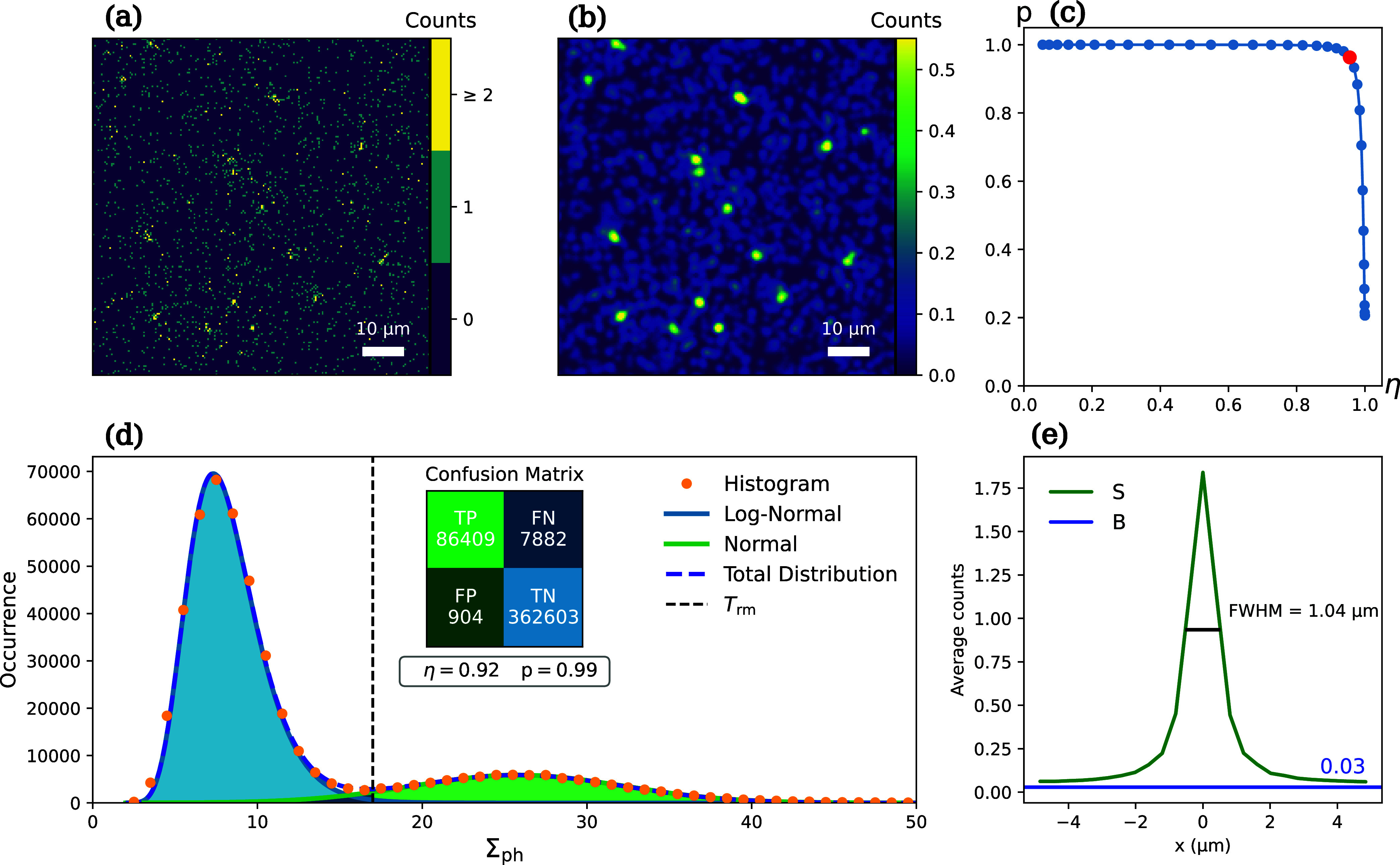
Data analysis for 30 keV electrons: (a) Zoom-in of a square subregion of the raw data frame; (b) Same subregion after applying a Gaussian filter. (c) purity *p* versus efficiency η curve, with the maximum *F*_1_-score indicated. (d) Histogram (orange dots) of photon counts within a circle of diameter *d*_ref_, centered on local maxima in the Gaussian-filtered image. The histogram is fit with a linear combination of Log-Normal and Normal distributions. The confusion matrix for binary classification is calculated with a threshold *T*_rm_= 17 counts, which optimizes the purity and is used to select events for computing the average PSF. (e) Cross section of the average PSF (S, green line) with corresponding FWHM = 1.04 μm, and the average background (B, blue line).

We can now use the fitted distributions to find a threshold that optimally discriminates between noise and single-electron detection events. For a given threshold, we calculate the confusion matrix, i.e., true (*T*) and false (*F*) positives (*P*) and negatives (*N*), which are defined as the integrals of the bodies and tails of the fitted distributions, as indicated by the shaded areas in Figure 2(d), and as detailed in the SI. The efficiency η = *TP*/(*TP* + *FN*) describes the probability that an electron is detected, while the purity *p* = *TP*/(*TP* + *FP*) gives the ratio of detected events that actually correspond to an electron. Choosing a classification threshold involves a compromise between η and *p*, as indicated in Figure 2c. It is a common choice to find the compromise by maximizing the *F*_1_-score, $F_{1}:=\frac{2\eta p}{\eta +p}$ . In our case, this maximization yields *F*_1_ = 0.96 for a threshold at Σ_ph_ = 15, which corresponds to an efficiency η = 0.96, and a purity *p* = 0.96, as indicated by the red dot in Figure 2c.

To further characterize the optical detection scheme, we sum up 89275 detection events with their local maxima superposed, yielding a proxy for the point-spread function (PSF) of the optical system. To minimize the influence of FP events on the PSF, we choose a threshold of Σ_ph_ = 17 (vertical dashed line in Figure 2d), corresponding to *p* = 0.99. The PSF cross section is shown in Figure 2e (S, green line), together with the average background (B, blue line), computed as the average across all pixels of the averaged background image. We obtain a full width at half-maximum FWHM = 1.04 μm, assuming linear interpolation between pixel values, see Figure 2e. To get an estimate of the spatial resolution of our detector at 30 keV, we also have to consider the random walk-off *d̃*_ic_ discussed previously. Adding them in quadrature, yields a resolution estimate of $\delta x=\sqrt{{\mathrm{FWHM}}^{2}+{\left(2{\mathrm{d̃}}_{\mathrm{ic}}\right)}^{2}}=2.3\mu m$

### Detection Characteristics as a Function of Electron Energy

Figure 3 illustrates the detector’s characteristics as a function of the electron energy *K*_in_. First, Figure 3a shows that the measured FWHM increases with *K*_in_, which is due to the increased size of the scintillation plume σ_G_. At energies below 20 keV the curve levels off, mainly due to the effective pixel size in the scintillator plane (0.4 μm). If we again combine this measurement with the simulated walk-off from Figure 1c, we obtain a resolution estimate of δ*x* = 0.9 μm at an electron energy of 17 keV.

**Figure 3:**
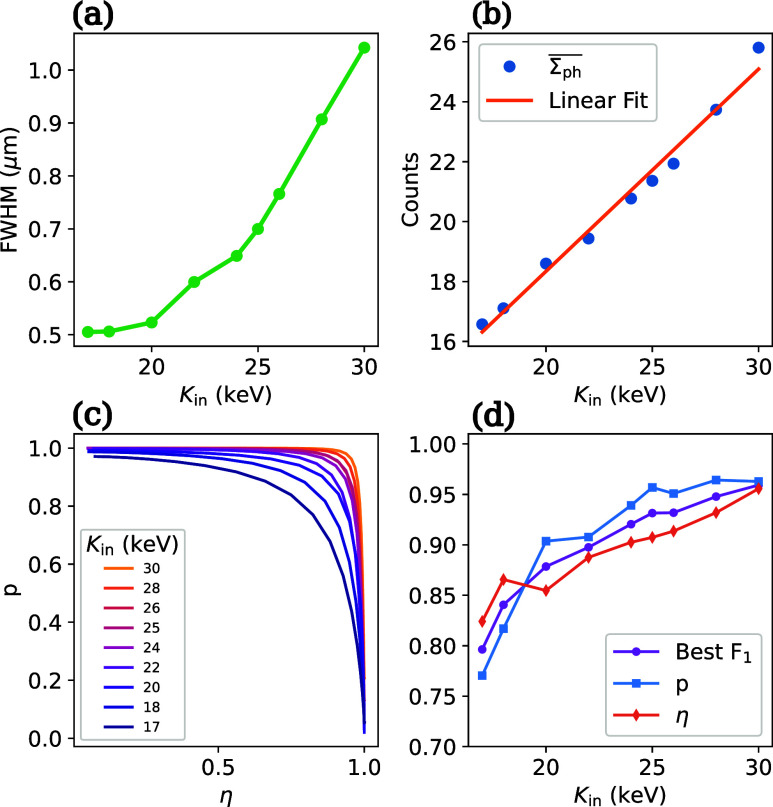
(a) Measured FWHM of the PSF as a function of energy. (b) Average photon counts per detected event as a function of energy (blue line, round markers) and linear fit (orange line). The linear fit has a slope of 0.67 photons/keV and an intercept of 4.8 photons. (c) purity-efficiency curves for electron energies between 17 and 30 keV. (d) Best *F*_1_-score and corresponding purity *p* and efficiency η as a function of electron energy.

This increased spatial resolution comes at the cost of a higher classification error due to the lower number of photons detected per incoming electron. Figure 3b shows the dependence of the mean photon number $\overset{®}{\Sigma_{\mathrm{ph}}}$ on *K*_in_. A linear fit yields a slope of 0.67 detected photons per keV, and an intercept of 4.8 photons, in good agreement with the measured $\overset{®}{\Sigma_{\mathrm{ph}}}$ of the background in the SI.

The lower number of photons per event at lower energies leads to slightly decreased efficiency and purity, as shown in Figure 3c. The best *F*_1_ score at each energy is shown in Figure 3d, along with the respective efficiency and purity. As an example, at *K*_in_ = 17 keV, we obtain *F*_1_ = 0.8. Note that in practice, a maximal *F*_1_ score does not necessarily represent the optimal condition for a specific experiment.

### Toward Electron Diffraction at Atmospheric Pressures

Lastly, we use our detector for diffraction studies in which the sample–detector distance has to be minimized. Figure 4 shows the diffraction pattern obtained with 30 keV electrons from a standard oriented gold crystal (Edge Scientific, EM-Tec TC1) usually employed for transmission electron microscopy calibration.

**Figure 4:**
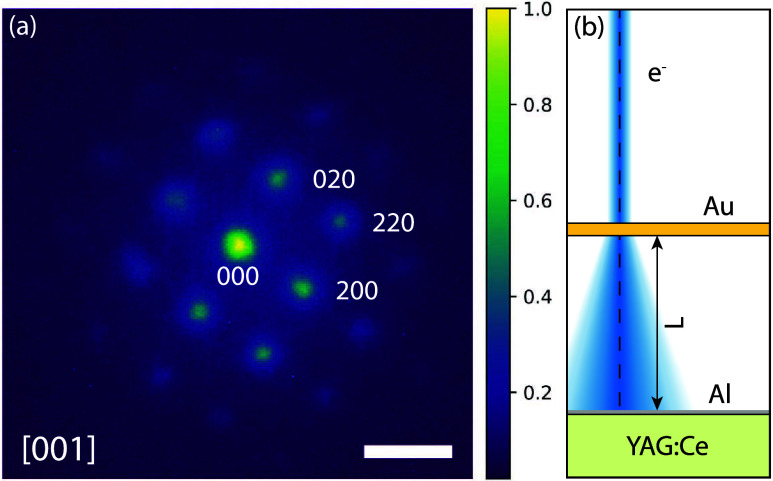
(a) Diffraction pattern of a [001]-oriented single gold crystal, obtained with a 30 keV electron beam. Scale bar: 15 μm in the detection plane. (b) Measurement scheme: The gold crystal (Au) is on a 300 mesh gold TEM grid (omitted in sketch) and placed on top of the YAG:Ce scintillator with a plastic spacer in between, leading to a sample-screen distance of *L* = 380 μm.

From the positions of the diffraction peaks, we calculate the distance between the crystal and the screen to be *L* = 380 μm, comparable to the mean free path length of 30 keV electrons in air (∼75 μm) or helium (∼800 μm).^17^ Fitting the diffraction orders with a Gaussian yields a FWHM of 4.2 μm. This is slightly larger than the resolution estimate δ*x*, likely due to finite coherence, electromagnetic stray fields, and mechanical vibrations in our setup, where the detector is mounted at a distance of 0.7 m below the objective lens of the SEM.^18^ Nevertheless, the distance between two diffraction orders can be determined with a precision much better than δ*x*. Specifically, using 2D Gaussians to determine the positions of the diffraction peaks, we can measure the distance between the zeroth diffraction order and the 020-peak with a precision 60 nm, given by the standard deviation from 25 measurements. This corresponds to an angular precision of 160 μrad, and a relative precision of Δ*g*/*g* of 4 × 10^–3^, where *g* is the lattice spacing.

## Discussion

We have demonstrated a single-electron detector with an estimated energy-dependent spatial resolution on the order of 1 μm in the energy range 17 keV–30 keV. The detector offers high efficiency and purity in distinguishing single-electron detection events from background noise. We have shown that the detector enables electron diffraction studies with a distance between the sample and the screen as short as 380 μm. This is on the order of the mean free path of 30 keV electrons in air (∼75 μm), and Helium (∼800 μm).^17^ Combined with vacuum-sealed electron guns,^19^ our detector thus enables diffraction studies at atmospheric pressures, complementing atmospheric scanning (transmission) electron microscopy.^17,19^ This potentially enables high-throughput studies and quality-control applications in which the samples no longer have to be transferred into vacuum. Importantly, we demonstrated that the narrow point-spread function of the imaging system enables high precision in localizing the diffraction orders. Specifically, we showed that we can determine the angle between diffraction orders with a precision of 160 μrad, and a relative precision of Δ*g*/*g* = 4 × 10^–3^. This is sufficient for identifying a material and its orientation, and enables high-precision miniature diffraction and spectroscopy applications.^20–23^ The relative precision could be improved by increasing the sample–detector distance, potentially enabling SEM-based high-precision strain measurements, which currently operate at a relative precision of ∼10^–4^.^24–26^

The use of a scintillator with custom optical detection setups enables experimental flexibility in terms of detector specifications. For example, our detector would be compatible with event-based cameras,^27^ which could enable fast acquisition at a low data acquisition rate. Considering the fast temporal response of the YAG:Ce scintillator material (rise time down to 1 ns, decay time down to 85 ns^28^), our detector can also be an excellent choice for time-resolved studies, especially when combined with fast camera technology, such as gated intensifiers or fluorescence lifetime imaging cameras.^29–31^ Future implementations might also use GAGG­(Ce) scintillators, which offer higher photon yield and material density, potentially leading to a higher signal-to-noise and a smaller detection plume, respectively.

The high resolution of our detector also lowers the demand for magnification in electron optical setups. This will enable more sensitive quantum electron optics experiments, such as ponderomotive electron wavefront shaping in a low-intensity limit.^18^ It can also benefit compact electron optics setups, such as tabletop low-energy TEMs.^32,33^

## Supplementary Material



## Data Availability

The data underlying this study are openly available in PHAIDRA, the repository for the permanent secure storage of digital assets at the University of Vienna, at 10.25365/phaidra.756.

## Notes

Collection efficiency of the optical system; Monte Carlo simulation of energy deposition in the scintillator; Image processing and event detection; Characterization of Background Noise; Binary classification of candidate detection events (PDF)
