# Supplementary material for: Low-Energy Single-Electron Detector with Submicron Resolution
Source: ACS Photonics. 2025 Dec 21;13(1):298–302. doi: 10.1021/acsphotonics.5c02404 (PMC12784400; doi:10.1021/acsphotonics.5c02404)
Supplement: Supplementary file 1 [file ph5c02404_si_001.pdf]

Supporting Information: Low-energy single-electron detector with sub-micron resolution

Luis Alfredo Ixquiac Méndez,[†,‡] Martino Zanetti,[†,‡] Tilman Kraeft,[†,‡] and Thomas Juffmann[*,†,‡]

† *University of Vienna, Faculty of Physics, VCQ, 1090 Vienna, Austria*

‡ *University of Vienna, Max Perutz Labs, 1030 Vienna, Austria*

E-mail: thomas.juffmann@univie.ac.at

Number of pages: 6

Number of tables: 0

Number of figures: 2

Collection efficiency of the optical system

To calculate the efficiency of the optical system, note that due to space constraints, a 45° mirror (Thorlabs PF10-03-P01P) is inserted in the optical path, and the camera is placed at 90° to the objective. The overall efficiency of the optical system is

$$\eta_L = \eta_{coll} \cdot (1 + R) \cdot \eta_{obj} \cdot \eta_{mirr} \cdot \eta_{T.L.} \cdot DQE$$

where η are respectively the collection efficiency ($\eta_{coll} = \frac{\Omega}{4\pi} \sim 0.31$, for NA=1.40), the objective transmission efficiency ($\eta_{obj} = 0.90$), the 45° mirror reflectance ($\eta_{mirr} = 0.90$) and

the transmittance of the tube lens ($\eta_{T.L.} = 0.97$). Furthermore, R=0.84 is the reflectance of the Aluminium layer deposited on the scintillator,[1] and DQE=0.8 is the detector quantum efficiency of the camera. All the parameters are estimated at the scintillation wavelength of 547 nm, resulting in $\eta_L = 0.38$.

Monte Carlo Simulation of energy deposition in the scintillator

The Monte Carlo simulation is performed using the CASINO simulation software.[2] The simulation models 7×10^5 electrons with kinetic energy of 30 keV impinging at a right angle on the Al coating surface on top of the scintillator. The incident electrons move in the $+z$ direction. For each electron, the simulation yields a trajectory of N points ($(x_i, y_i, z_i) : i \in \{1, \ldots, N\}$), with corresponding kinetic energies E_i. For each electron trajectory, we calculate the center of deposited energy as $r_\mathrm{c} = (x_\mathrm{c}, y_\mathrm{c}) \coloneqq \frac{\sum_{i=2}^{N}(x_i,y_i)\cdot(E_{i-1}-E_i)}{E_1}$

Figure S1a(b) shows the deposited energy, integrated along the z(y) axis. The distribution represents the average across all simulated trajectories, normalized to the total energy. Note that the distributions of individual electrons are aligned with respect to their centers of deposited energy r_c. The solid lines denote the areas that contain 25, 50, 68/75, and 90% of the total deposited energy in a/b, respectively. These calculations are repeated for different energies, providing the data for the purple line in Figure 1c.

Figure S1c shows a histogram of the distances between the coordinate of the incoming electrons and their simulated r_c for two different energies. The median of the distributions is indicated by the vertical dashed lines. These calculations are repeated for different energies, providing the data for the blue line in Figure 1c.

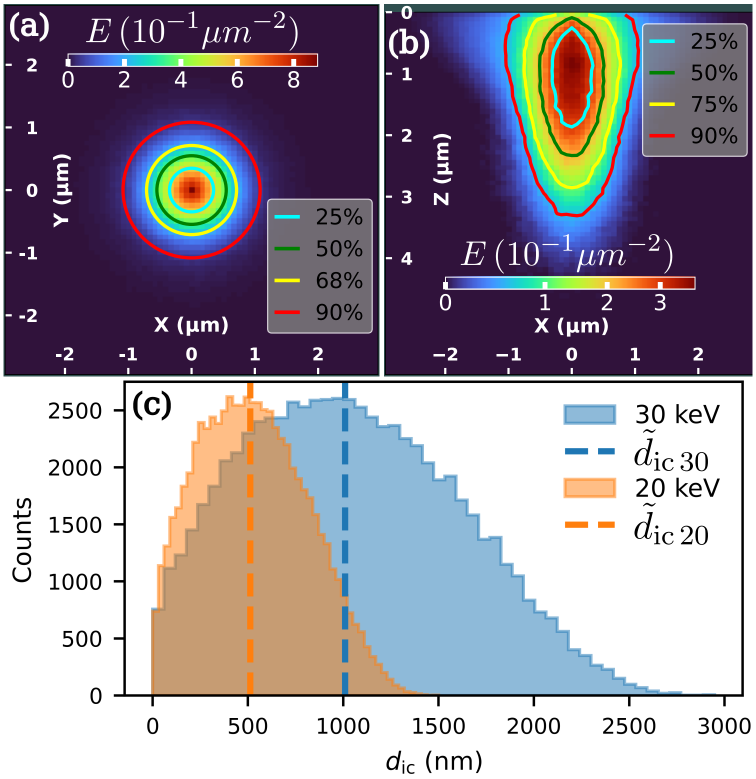

Figure S1: Monte Carlo simulation: (a) and (b) normalized x-y and y-z projections of the spatial distribution of energy deposited by the electrons on the scintillator. Electron trajectories used in the computation are aligned with respect to their centre of deposited energy $r_{\text{coel}} = (x_{\text{c}}, y_{\text{c}})$. (c) Distributions of d_{ic} (distance of the center of deposited energy from the entrance point) for 30 keV electrons (blue, right) and 20 keV electrons (orange, left). The dotted lines mark the medians of each distribution.

Image processing and event detection

Image analysis is performed on a circular region of interest (ROI) with a diameter of 320 μm.

To obtain a background image, we average 200 images with the electron gun off. From the same set of images, we calculate the standard deviation in background counts on each pixel. Pixels above the 99.99 th percentile of the mean and standard deviation are considered dead pixels. After background subtraction, Gaussian filtering is performed. We use the radius of the distribution of simulated deposited energy (σ_G, see Figure 1c) as the width of the Gaussian, defined on a circular kernel of diameter $2\,(\mathrm{round}(2\sigma_G/(0.4\,\mu\mathrm{m}))) + 1\,\mathrm{px}$.

After finding all local maxima, we reject those that are closer than $d_{ref}/2$ to a dead pixel. Similarly, if two local maxima are at a distance smaller than d_{ref}, the weaker is rejected. Furthermore, we reject spurious detection events, defined as maxima with more than 7 counts. These are statistically very unlikely from single-electron events.

Characterisation of Background Noise

Performing the above procedure on the data set of background images, we obtain the histogram with the photon number distribution of dark counts shown in Figure S2(a). We found empirically that they follow a log-normal distribution. This is a consequence of the Gaussian smearing and peak finding that is done before calculating Σ_{ph}. We verified this with simulated Poissonian background distributions, for which our algorithm also yields a log-normal distribution (see S2(b)). Despite the good qualitative fit of the log-normal distribution, we note that it slightly overestimates the counts in the tail of the background distribution. Finding a better fit would yield a slightly lower number of false positives and improve the detector specifications we report in our manuscript.

The read noise is calculated on a set of 740 images taken with the gun off across the whole chip of the camera. We calculate the standard deviation in the number of detected photons for each pixel throughout the images, and calculate the average standard deviation

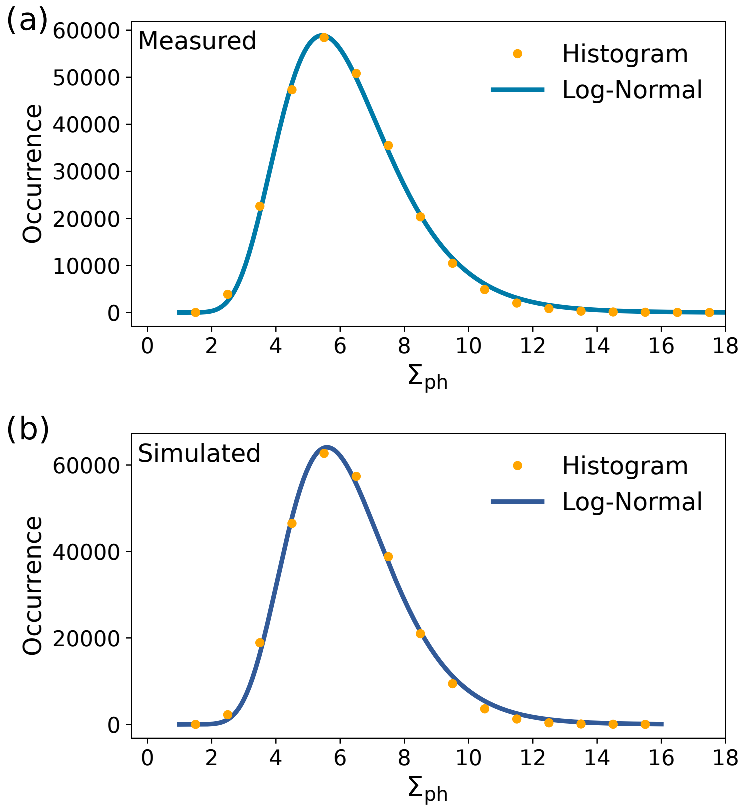

Figure S2: Background photon number distribution: (a) experimental histogram (orange dots) of candidate events with Σ_{ph} photons contained in a circle of diameter $d_{ref} = 5.2\,\mu$m centered at local maxima coordinates of gaussian filtered images of dark background, and Log-Normal fit of the distribution. (b) simulated histogram using the specifications of our camera.

across all pixels. This yields 0.13 counts rms.

Binary classification of candidate detection events

With the electron gun on, we obtain the histogram in Figure 2d. Binary classification requires defining a threshold T_{rm} of photon counts to select single-electron detection events ($\Sigma_{ph} \geq T_{\mathrm{rm}}$) and reject the dark counts ($\Sigma_{ph} < T_{\mathrm{rm}}$).

The true positives (TP), false positives (FP), true negatives (TN), and false negatives (FN) are defined on the fitted distributions and are given by

$$TP := \int_{T_{\mathrm{rm}}}^{\infty} N(x)dx \quad FP := \int_{0}^{T_{\mathrm{rm}}} N(x)dx$$

$$TN := \int_{0}^{T_{\mathrm{rm}}} L_{\mathrm{N}}(x)dx \quad FN := \int_{T_{\mathrm{rm}}}^{\infty} L_{\mathrm{N}}(x)dx$$

where N and L_{N} are, respectively, the fitted normal and log-normal distributions. These four numbers define the confusion matrix of the binary classification, which is shown in Figure 2d.

References

(1) Corporation, K. Filmetrics Reflectance Calculator. 2025; `https://www.kla.com/products/instruments/reflectance-calculator`, Accessed: 2025-09-08.

(2) Demers, H.; Poirier-Demers, N.; Réal Couture, A.; Joly, D.; Guilmain, M.; de Jonge, N.; Drouin, D. Three-dimensional electron microscopy simulation with the CASINO Monte Carlo software. *Scanning* **2011**, *33*, 135–146.
